# Different Responses of Bacterial and Archaeal Communities in River Sediments to Water Diversion and Seasonal Changes

**DOI:** 10.3390/microorganisms9040782

**Published:** 2021-04-08

**Authors:** Jiali Lv, Yangdan Niu, Ruiqiang Yuan, Shiqin Wang

**Affiliations:** 1 School of Environment and Natural Resources, Shanxi University, Taiyuan 030006, China; lvjiali19@mails.ucas.ac.cn (J.L.); niuyangdan@tongji.edu.cn (Y.N.); 2 Key Laboratory of Agricultural Water Resources Research, Innovation Academy for Seed Design, Center for Agricultural Resources Research, Institute of Genetics and Developmental Biology, Chinese Academy of Sciences, Shijiazhuang 050021, China; sqwang@sjziam.ac.cn; 3 Sino-Danish College of University of Chinese Academy of Sciences, Beijing 101408, China

**Keywords:** archaea, bacteria, 16SrRNA high-throughput sequencing, water transfer, seasonal changes, river sediments

## Abstract

In recent years, different responses of archaea and bacteria to environmental changes have attracted increasing scientific interest. In the mid-latitude region, Fen River receives water transferred from the Yellow River, electrical conductivity (EC), concentrations of Cl^−^ and Na^+^ in water, total phosphorus (TP), and Olsen phosphorus (OP) in sediments were significantly affected by water transfer. Meanwhile, temperature and oxidation-reduction potential (ORP) of water showed significant seasonal variations. Based on 16S rRNA high-throughput sequencing technology, the composition of bacteria and archaea in sediments was determined in winter and summer, respectively. Results showed that the dominance of bacterial core flora decreased and that of archaeal core flora increased after water diversion. The abundance and diversity of bacterial communities in river sediments were more sensitive to anthropogenic and naturally induced environmental changes than that of archaeal communities. Bacterial communities showed greater resistance than archaeal communities under long-term external disturbances, such as seasonal changes, because of rich species composition and complex community structure. Archaea were more stable than bacteria, especially under short-term drastic environmental disturbances, such as water transfer, due to their insensitivity to environmental changes. These results have important implications for understanding the responses of bacterial and archaeal communities to environmental changes in river ecosystems affected by water diversion.

## 1. Introduction

Bacteria and archaea are important elements of a community of microorganisms, which participate in and influence the process of material circulation and energy transmission in nature [1,2,3]. In river sediments, bacteria and archaea can dominate the nitrogen cycle and the corresponding energy transfer process [4,5,6]. Bacteria are widely distributed in all corners of the earth [1], but in extreme environments, the abundance and diversity of bacteria decreases, while archaea become the main microorganisms [7,8], and the proportion of extremophiles increases significantly. Bacteria and archaea belong to two evolutionary branches that are very distinct from each other, and different genes lead to different responses of bacteria and archaea to different conditions [9,10]. For example, ammonia-oxidizing archaea (AOA) and bacteria (AOB) react differently to nitrogen concentrations [11]. At the same time, bacteria and archaea have different reactions to different heavy metal concentrations [12,13,14,15], drought sensitivity [2,16], and many other conditions. In addition, archaea have a special membrane structure, causing some difficulties to use ATP, and therefore, it would enable itself to extract its energy from a natural pH gradient [3,17,18,19]. Thus, early on, it was believed that archaea had been all extremophiles, which means they could thrive in high temperature, high salinity, low or high pH, absolute anaerobic or combinations, and that maybe something about their physiology made them poor competitors with bacteria in more normal niches. What turned the tide was the PCR amplification and Sanger-based sequencing of 16S rRNA genes present in environmental samples, which was the beginning of finding archaea in nonextreme environments, and many new archaeal and bacterial lineages (including phyla) have been discovered.

Recently, comparative studies of archaea and bacteria in nonextreme environments, including wetlands [20,21], coastal zones [22] lakes [23,24], and soil [25,26,27] have become more and more popular. In different habitats, bacteria and archaea showed different community structures and compositions. Despite some fluctuating environmental conditions, many microbial taxa displayed significant seasonal changes [28]. The diversity of bacterial communities in coastal wetlands was higher than that of archaeal communities, and the temperature was the main factor driving the seasonal changes of bacterial communities [29]. Archaeal communities in the Bay of Banyuls area were strongly influenced by terrestrial sources, but changes in marine conditions played a more important role in the construction of bacterial communities [28]. Bacteria and archaea in the Alps showed obvious seasonal variation rules, and the abundance of archaea reached a peak in cold spring and winter [30]. In general, the abundance of bacteria was much higher than that of archaea, while in arid saline land, the abundance of archaea was much higher than that of bacteria [31]. By observing the bacterial and archaeal communities in different habitats, it was found that the bacterial communities often changed to different degrees with the change of topography and landform and the dry and wet seasons, while the changes of archaeal communities were not obvious [32,33]. However, the change of archaeal community might be very significant in rivers or estuaries affected by sewage discharge [6], the abundance of archaea is sometimes greater than that of bacteria [5], and the environmental factors affecting bacteria and archaea are also completely different [13]. These factors suggest that bacteria and archaea respond obviously differently to environmental changes caused by seasonal changes and human activities.

Natural changes such as seasonal variations tend to change slowly and put less stress on bacteria and archaea. In contrast, large-scale human activities can often change the environment in a short period of time, and drastic environmental changes put bacteria and archaea under greater environmental stress. The exploration of the response of bacteria and archaea to environmental changes caused by seasonal changes and human activities could lead scientists to a deeper understanding of bacteria and archaea. However, studies in this field have explored the different responses of bacteria and archaea in rivers to large human activities, such as urban drainage [34] and reservoir construction [35], while insufficient attention has been paid to the effects of water transfer projects.

Water diversion projects are common efforts to balance regional water resources. However, water diversion projects, especially inter-basin water diversion projects, may lead to a high degree of spatial and temporal heterogeneity of the ecological environment in the water receiving area and have a profound impact on the bacterial and archaeal communities [36,37]. At the same time, most water diversion projects are located in the mid-latitude region with a concentrated population and arid/semi-arid climate. The heterogeneity is further deepened by the distinct seasonal changes. Previous studies have shown that bacteria in water bodies and sediments affected by water diversion have significant spatiotemporal changes [38,39,40,41,42]. However, the different responses of bacterial and archaeal communities in river sediments affected by both water diversion and seasonal changes are currently less known.

Based on 16S rRNA high-throughput sequencing technology, this paper determined bacterial and archaeal community compositions in river sediments influenced by inter-basin water transfer in a mid-latitude region, and discussed the changes of river sediments bacterial and archaeal communities under the anthropogenic water transfer activity and natural seasonal changes, comparatively analyzing different responses between the bacterial and archaeal communities. This study aimed to contribute to the understanding of bacteria and archaea communities in changing environments.

## 2. Materials and Methods

### 2.1. Study Area and Sampling Site

**Figure 1:**
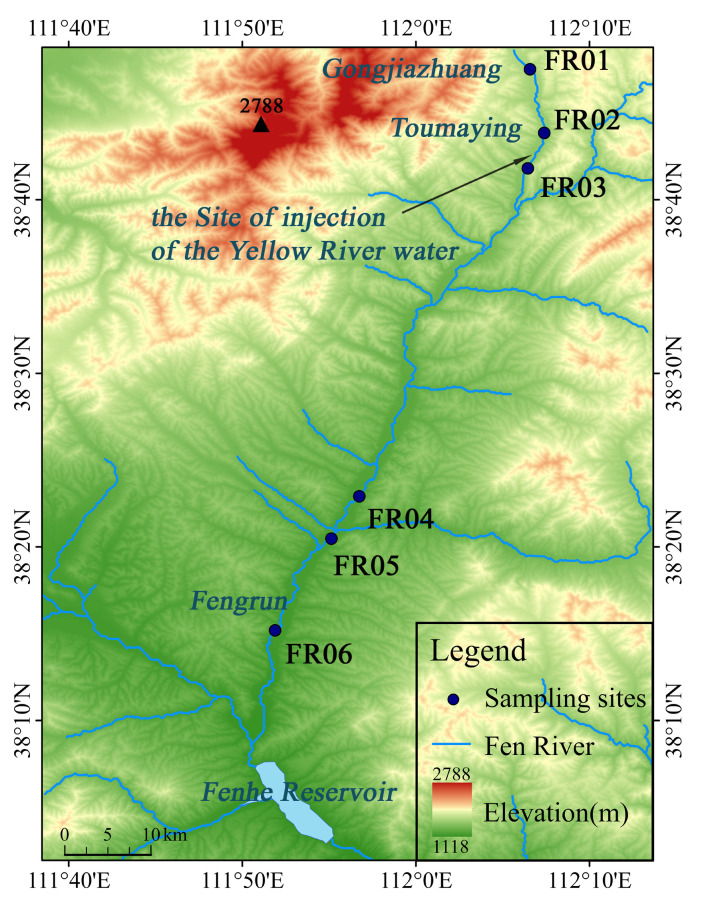
Map of the study area and sampling sites.

The Fen River, 710 km in length, is the second-largest tributary of the Yellow River in China (Figure 1). The study area located in 38°48′~38°15′ N, 111°52′~112°07′ E, covering the reach from the river head (Gongjiazhuang) till Fengrun, with a length of around 80 km. The research area has a temperate monsoon climate with dry cold winters and wet hot summers. The annual average temperature is about 7 °C, with an average temperature of −9 °C in January and 21 °C in June. The annual rainfall is 380 mm to 500 mm, about 70% of which occurs from June to September. The study river reach has been receiving an injection of 320 million m^3^/a from the Yellow River water in Toumaying since 2003. The water diversion is usually suspended in February, March, August, and September.

The sampling site of FR01 (Gongjiazhuang) is about 1 km away from the source of the Fen River (Figure 1). The site of FR02 is 100 m upstream of the Yellow River water injection point (Toumaying). The sampling site of FR03 is 4.5 km downstream of Toumaying, with no tributary. The site of FR04 is located about 50 km downstream of FR03. Multiple tributaries are fed in the river reach between FR03 to FR04, although their total runoff is relatively limited. The sampling site of FR05 is located about 6 km downstream of FR04, followed by FR06 about 15 km downstream.

### 2.2. Sample Collection and Measurements

Samples were collected from six sampling sites in winter (January) and summer (June) of 2017, respectively. Before sampling, physicochemical parameters, such as water temperature, electrical conductivity (EC), oxidation-reduction potential (ORP), and total dissolved solids (TDS) of the river water, were measured (HORIBA U-51). Then, three replicate samples were collected at each site. Water samples below 20 cm from the water surface in the center of the river were collected and loaded into three 60 mL high-density polyethylene bottles. About 1 kg sediment samples were collected and put into sterile zip-lock bags. These samples were stored in a laboratory refrigerator at 4 °C until physicochemical analysis.

River sediment samples of 1–2 cm below the surface of sediments were homogenized with five surficial sediment locations collected in a 1 × 1 m sample area. Three replicate samples were collected at each site and then sealed in 10 mL aseptic centrifugal tubes and placed in dry ice. All sediment samples were frozen with dry ice immediately after collection, then were stored at −80 °C until DNA extraction [43].

The contents of Ca^2+^, Mg^2+^, Na^+^, and K^+^ in water samples were determined using ICP-OES (PerkinElmer 5300DV), and the contents of Cl^−^, SO_4_^2−^ and NO_3_^−^ were determined using an ion chromatograph (Dionex ICS-900). The contents of HCO_3_^−^ were measured by titration on the sampling day. The particle size (PS) of river sediment samples was analyzed by a laser particle size analyzer (Mastersizer 2000) [44]. In addition, total nitrogen (TN) and alkaline nitrogen (AN) were measured by the Kjeldahl method and alkaline solution diffusion method, respectively [45]. The sodium hydroxide fusion method was used to measure total phosphorus (TP) and total potassium (TK) [46]. Sodium bicarbonate extraction method (Olsen) and ammonium acetate extraction measurement were common methods for the determination of Olsen phosphorus (OP) and available potassium (AK), respectively [47,48]. Soil organic matter (SOM) content was determined by H_2_SO_4_—K_2_Cr_2_O_7_, the external heating oxidation method [49]. The instruments used include an atomic absorption spectrometer (Zeenit of Jena, Germany 700 p), a spectrophotometer, and the Kjeldahl apparatus.

### 2.3. DNA Extraction and PCR Amplification

Total DNA was extracted from each replicate sample using FastDNA^®^ Spin Kit for Soil (MP Biomedicals, CA, USA) according to the manufacturer’s instruction. The DNA extract was checked on 1% agarose gel, and DNA concentration and purity were determined with NanoDrop 2000 UV–VIS spectrophotometer (Thermo Scientific, Wilmington, DC, USA).

The V3–V4 hypervariable regions of the bacteria and archaea 16S rRNA gene were amplified with primers 338F (5′–ACTCCTACGGGAGGCAGCAG–3′)/806R (5′–GGACTACHVGGGTWTCTAAT–3′) [50] and 524F10extF (5′–TGYCAGCCGCCGCGGTAA–3′)/Arch958RmodR (5′–YCCGGCGTTGAVTCCAATT–3′) [51] by an ABI GeneAmp^®^ 9700 PCR thermocycler (ABI, CA, USA), respectively. PCR was performed with the following program: 3 min of denaturation of template DNA at 95 °C, 27cycles of 30 s at 95 °C, 30 s for annealing at 55 °C, 45 s for elongation at 72 °C, and a final extension at 72 °C for 10 min.

### 2.4. Illumina MiSeq Sequencing

Amplicons from each PCR sample were normalized to equimolar amounts and sequenced using 468 bp chemistry on a MiSeq PE300 platform (Illumina, San Diego, CA, USA) at Majorbio Biopharm Technology Co., Ltd. (Shanghai, China). The sequencing data were submitted to the NCBI Sequence Read Archive database under accession number SUB8145997. Subsequently, 16S rRNA sequencing data were processed using the Mothur MiSeq pipeline [52]. Then, 16S rRNA V4–V5 region genes were amplified in triplicate from each pooled RNA sample to investigate the population of bacteria and archaea, using relevant paired primers, respectively. Using fastp (https://github.com/OpenGene/fastp (accessed on 19 June 2017), version 0.20.0), software quality control was carried out on the original sequencing sequence [53]. FLASH (http://www.cbcb.umd.edu/software/flash (accessed on 19 June 2017), version 1.2.7) software was used for Mosaic [54]. UPARSE software (http://drive5.com/uparse/ (accessed on 6 March 2018), version 7.1), was used according to 97% operational taxonomic units (OTUs) on a sequence of similarity clustering [55,56]. Classifier (http://rdp.cme.msu.edu/ (accessed on 6 March 2018), version 2.2) was used to annotate the species classification of each sequence, and the comparison was made to the Silva 16S rRNA database (V128), with the comparison threshold being 70% [56].

The sequencing results of the sampling sites FR01–FR06 in winter and summer were labeled as W1–W6 and S1–S6, respectively.

### 2.5. Statistical Analysis

The physicochemical characteristics of river water and sediment were analyzed by t-test with SPSS Statistics, Version 20.0 (International Business Machines Corporation, NYC, USA). To analyze the bioinformation of the high-throughput data, quantitative insights into microbial ecology (QIIME), Version 1.9.1 was used to classify the sequences into operational taxonomic units (OTUs) using a 97% identity threshold [57]. The α-diversity, including Sobs, Chao, Shannon, and Coverage indexes, were calculated using the Mothur [58].

Principal component analysis (PCA) was conducted for environmental indicators by Origin, Version 2018 (OriginLab Corporation, Northampton, MA, USA). The microbial composition bar was mapped at the gene level using R, Version 3.6.3 (Microsoft, Redmond, WA, USA) [59]. The “pheatmap” package in R, Version 3.6.3 (Microsoft, Redmond, WA, USA) was employed to display the correlation between environmental factors and species visually. Heat map was based on the relationship between strains with relative abundance greater than 1% and environmental factors. Spearman correlation coefficient between these bacteria/archaea and environmental factors was calculated, and hierarchical clustering of environmental factors and taxa was conducted according to the correlation coefficient [60]. Wilcoxon rank-sum test method was used for hypothesizing to assess the significance level of species abundance differences and obtaining species with significant differences between groups with a double-tail test at the classification level of genes. The fdr multiple test calibration method was used to calibrate *p*-value with a confidence interval of 0.95 [61].

To study the similarity or difference relationship of different sample community structures, cluster analysis was performed on the sample community distance matrix. quantitative insights into microbial ecology (QIIME), Version 1.9.1 used the Bray–Curtis distance algorithm to calculate the beta diversity distance matrix and then used the upgma function in the “phangorn” package in R, Version 3.6.3 (Microsoft, Redmond, WA, USA) to sample clustering and the par function to draw the tree graphically [62]. The network analysis was constructed by calculating the correlation between taxa using Networkx [63]. The nodes in the network diagram are species nodes, and the connecting lines show that the correlation coefficient between species and species is greater than 0.8. Parameters in statistical analysis were introduced in the Supplementary Materials. Evolutionary trees were constructed using Mega (version 10.0, https://www.megasoftware.net/ (accessed on 23rd April 2019) by sequences corresponding to taxonomic information according to the maximum likelihood (ML) method, and the phylogenetic relationships of species were presented in the form of a ring diagram using the R, Version 3.6.3 (Microsoft, Redmond, WA, USA) [64]. The smallest taxonomic unit is the genus.

In total, 3,145,879 high-quality 16S rRNA sequences were generated for 72 samples. After subsampling each sample to an equal sequencing depth (above 30,000 reads per sample) and clustering, 7603 operational taxonomic units (OTUs) at 97% identity were obtained, with the number of OTUs ranging from 1193 to 3988 per sample. The Good’s coverage for the observed OTUs was 98.67 ± 0.15% (mean ± s.e.m.), and the rarefaction curves showed clear asymptotes (Figure S1), which together indicate a near-complete sampling of the community.

## 3. Results and Discussion

### 3.1. Physicochemical Features of River Water and Sediment

Significant variations of physicochemical parameters of the river water and the sediment caused by changing seasons and water transfer were found. From January to June, the temperature of the river water increased, and ORP decreased significantly (*p* < 0.01). The average increase of EC and TDS in the water-receiving reach was about 2.5 times the normal river reach. Particularly, concentrations of Cl^-^ and Na^+^ increased by 1985% and 1725% on average (Table 1).

Compared with winter (January), PS, AN, and OP of the sediments increased by 126%, 52%, and 28%, respectively, in summer (June), while SOM and AK decreased by 28% and 16%, respectively. Compared with the normal river reach, the contents of most nutrient salts in the water-receiving reach sediments visibly increased, with an increase of 156% for AN, 125% for AK, 89% for OP, 88% for TN, and 58% for SOM in January, while the contents of most nutrient salts in the water-receiving reach sediments decreased by 44%, 54%, and 55% for TN, AN, and OP, respectively, in June. On average, PS in the water-receiving reach decreased by 51% than that in the normal river reaches (Table 1).

**Figure 2:**
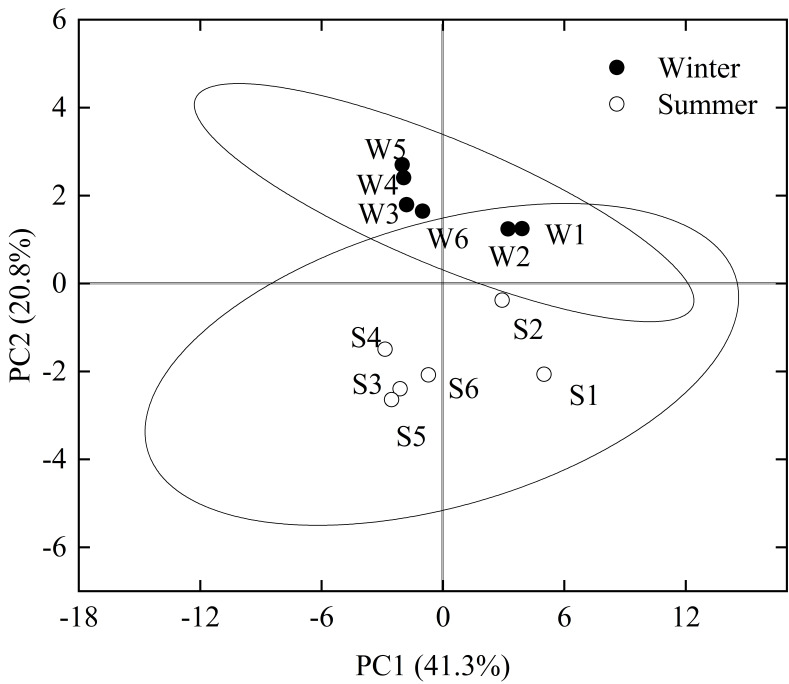
Result of the principal component analysis of physicochemical parameters of river water and sediments. Here, 95% confidence ellipses of sampling groups are shown. The sequencing results of the sampling points FR01–FR06 in winter and summer were labeled as W1–W6 and S1–S6, respectively.

Two principal components were recognized based on all physicochemical parameters and the principal component analysis (Figure 2). The samples of the normal river reach and the water-receiving reach were separated by the line of x = 0, while the samples of winter and summer were separated by the line of y = 0. Therefore, the first principal component (PC1) could reflect the influence of water diversion on the water environment of the Fen River, and the second principal component (PC2) could reflect the influence of seasonal changes on the water environment of the Fen River. According to the compositions of PC1 and PC2, environmental factors (physicochemical parameters) can be divided into three categories, i.e., (1) factors mainly relating to seasonal changes, including water temperature and ORP; (2) factors mainly relating to the water diversion, including EC, TDS, Cl^-^, Mg^2+^, K^+^, Na^+^, Ca^2+^, TP, and OP; and (3) other factors not obviously influenced by the changing seasons and water diversion.

### 3.2. Overview of Bacterial and Archaeal Communities

The number of sequences used for analyses is more than 30,000. The coverage index of all samples is above 97%. The sample sequence information can fully represent the sample population (Table 2). At the same time, the rarefaction curve of the Shannon index tended to be flat, indicating that the sequencing results could reasonably characterize the diversity of bacteria and archaea.

Overall, the bacterial community was 672% more than the archaeal community (Table 2). Affected by water transfer, the number of bacterial and archaeal OTUs in winter increased by 6.7% and 18.8%, compared to that of summer. Contrasting bacteria and archaea, seasonal changes caused a large change in the number of OTUs in the archaeal community, while that of bacteria changed little. This result was reasonable because seasonal changes in environmental factors could cause microbial biomass in natural river sediments to be higher in winter than in summer [65]. Seasonal changes in water temperature (*p* < 0.001), ORP (*p* < 0.001) and NO_3_^−^ (*p* < 0.05) were significant in the study area (Table 1). At the same time, there were obvious seasonal changes in the contents of SOM, potassium, nitrogen, and phosphorus in sediments (Table 1).

In total, the bacterial community was 111% more diverse than the archaeal community (Table 2). In addition, seasonal variations had little effect on the spatial heterogeneity of bacterial diversity, but rather affected the average level of bacterial diversity in the study area. Bacterial diversity was high in summer and low in winter. The opposite effect of seasonal variation was observed for archaea. The spatial heterogeneity of archaeal diversity was significantly greater in winter than in summer, and the average level of diversity of archaea in the study area changed little. The response of bacteria and archaea to water transfer was equally opposite. After water transfer, the diversity of bacteria increased by 2.7% and 7.4% in winter and summer, respectively, and the diversity of archaea decreased by 12.54% and 7.7% in winter and summer, respectively.

For bacteria, a total of 53 phyla, 123 classes, 267 orders, 356 families, and 1057 genera were identified. In summer, the amounts of species with their relative abundance greater than 1% was 44, while in winter it was 33. As for archaea, a total of 13 phyla, 25 classes, 32 orders, 36 families, and 58 genera were identified in the samples. There were 17 genera with relative abundance greater than 1% in summer, and the number was 16 in winter. According to distribution ranges, the abundance, and stability of the bacteria and archaea in sediments in different seasons, the taxa of bacteria and archaea with the highest abundance were defined as the core flora in this study [66,67].

**Figure 3:**
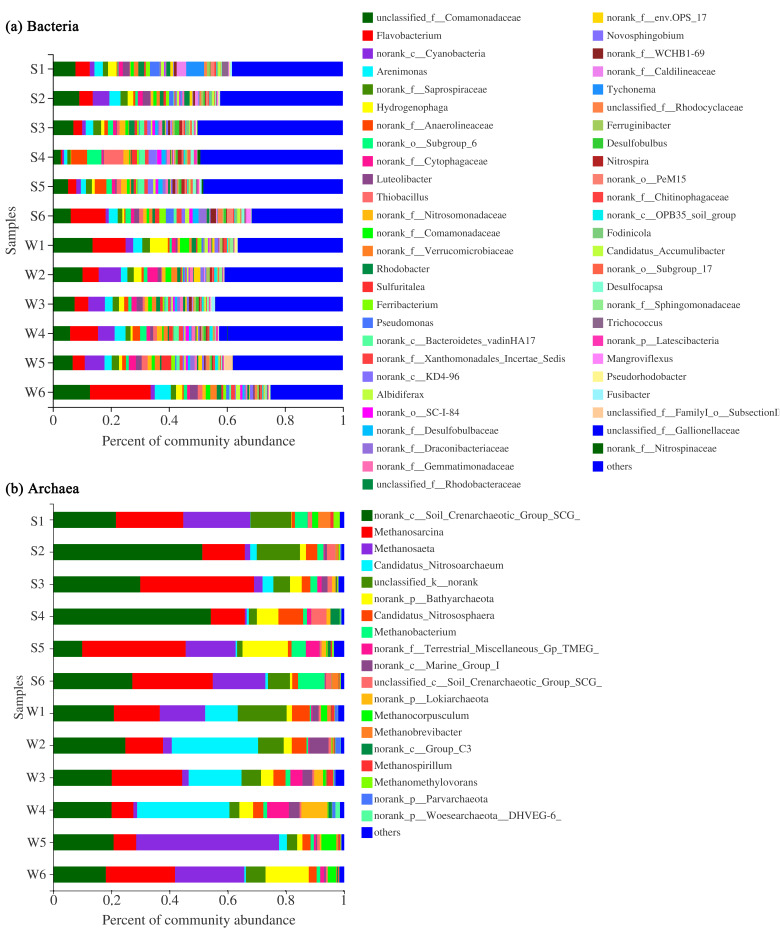
The microbial composition of bacteria (**a**) and archaea (**b**). The smallest taxonomic unit is the genus. The relevant abundance of taxa less than 1% were classified as others. The sequencing results of the sampling points FR01–FR06 in winter and summer were labeled as W1–W6 and S1–S6, respectively.

In winter, Comamonadaceae was the most abundant family, accounting for 5.9–13.9% of the total number of bacteria in each sample (Figure 3a), followed by *Flavobacterium* (4.5–21.3%), Cyanobacteria (1.4–6.7%), *Arenimonas* (2.2–5.6%), *Hydrogenophaga* (1.8–6.3), and Subgroup_6 (1.7–2.7%). In summer, Comamonadaceae was the most abundant family, accounting for 2.9–9.1% of the total number of bacteria in each sample, followed by *Flavobacterium* (2.8–12.2%), *Arenimonas* (1.1–4.0%), Saprospiraceae (1.1–4.0%), Anaerolineaceae (1.3–15.4%) and Subgroup_6 (1.9–4.9%). These bacteria constituted the core flora of the bacterial community in the river sediment. The dominant position of the core bacterial flora decreased slightly. The average relative abundance of the core bacterial flora in the normal river reach was about 33.9% and 21.5% for winter and summer, and that was about 29.4% and 20.4% in the water-receiving reach, respectively.

In winter, the core flora of the archaea communities consists of SCG (17.9–24.9%), *Methanosaeta* (1.2–49.1%), *Nitrosoarchaeum* (0.5–31.9%), *Methanosarcina* (7.5–24.0%), Bathyarchaeota (1.9–15.3%), *Candidatus_Nitrososphaera* (2.7–6.1%), and Marine_Group_I (0.4–6.6%) (Figure 3b). In summer, the core flora consists of SCG (9.9–54.6%), *Methanosarcina* (11.6–39.2%), *Methanosaeta* (0.6–22.8%), Bathyarchaeota (0.8–15.7%), *Methanobacterium* (1.3–9.2%), and *Candidatus_Nitrososphaera* (1.0–7.8%). The dominant position of the core archaea flora in sediments of the river increased slightly. The average relative abundance of the core archaea flora in the normal river reach was about 79.2% and 75.6% in winter and summer, respectively. Additionally, that was about 89.3% and 83.8% in the water-receiving reach in winter and summer, respectively.

### 3.3. Different Responses of Bacterial and Archaeal Core Floras to Water Diversion

**Figure 4:**
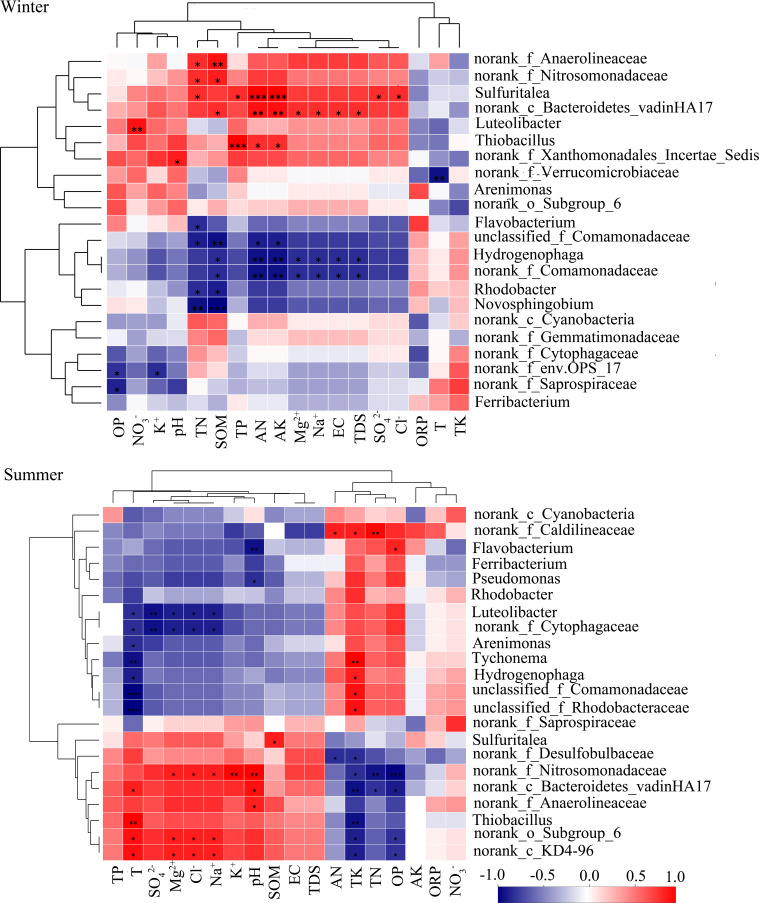
Heat map of environmental factor correlations of bacteria. The significant level was symbolled as: *: *p* ≤ 0.05, **: *p* ≤ 0.01, ***: *p* ≤ 0.001. Red represented positive correlation, blue represented negative correlation, the darker the color, the greater the correlation. The range of positive and negative correlation coefficients is −1 to 1. TP: total phosphorus; T: temperature; SOM: soil organic matter; EC: electrical conductivity; TDS: total dissolved solid; AN: alkaline nitrogen; ORP: oxidation-reduction potential; PS: particle size; TN: total nitrogen; OP: Olsen phosphorus; TK: total potassium; AK: alkaline potassium.

Compared to other bacteria, Anaerolineaceae, Bacteroidetes_vadinHA17, and *Thiobacillus* were better adapted to the sediment environment after water diversion. Anaerolineaceae and Bacteroidetes_vadinHA17 had similar correlations with environmental factors, and they clustered into similar clusters (Figure 4). These two bacterial taxa played an important role in the degradation of carbohydrates and other cellular materials in methanogenic biological systems [68,69]. Thus, they survived well in hypoxic or anoxic environments and could grow well in response to environmental changes by water transfer. Among them, the abundance of Anaerolineaceae increased in summer and became sub-dominant bacteria at FR04 (5.37%) and FR05 (3.88%) (Figure 3a). In summer, the relative abundance of *Thiobacillus* rose in FR04 (from 1.32% to 6.76%). The abundance of *Thiobacillus* was significantly negatively correlated with TK (Figure 4). Recent studies have demonstrated that *Thiobacillus* is one of the few bacteria capable of efficient denitrification at high potassium concentrations, thus validating results in this paper [70]. The TK content in the sediments of the water-receiving reach was 16% lower than that in the normal river reach, and the TK content in FR04 was the lowest. Thus, the abundance of *Thiobacillus* was significantly increased. In addition, the abundance of Xanthomonadales and SC-I-84 in the receiving river reach increased.

**Figure 5:**
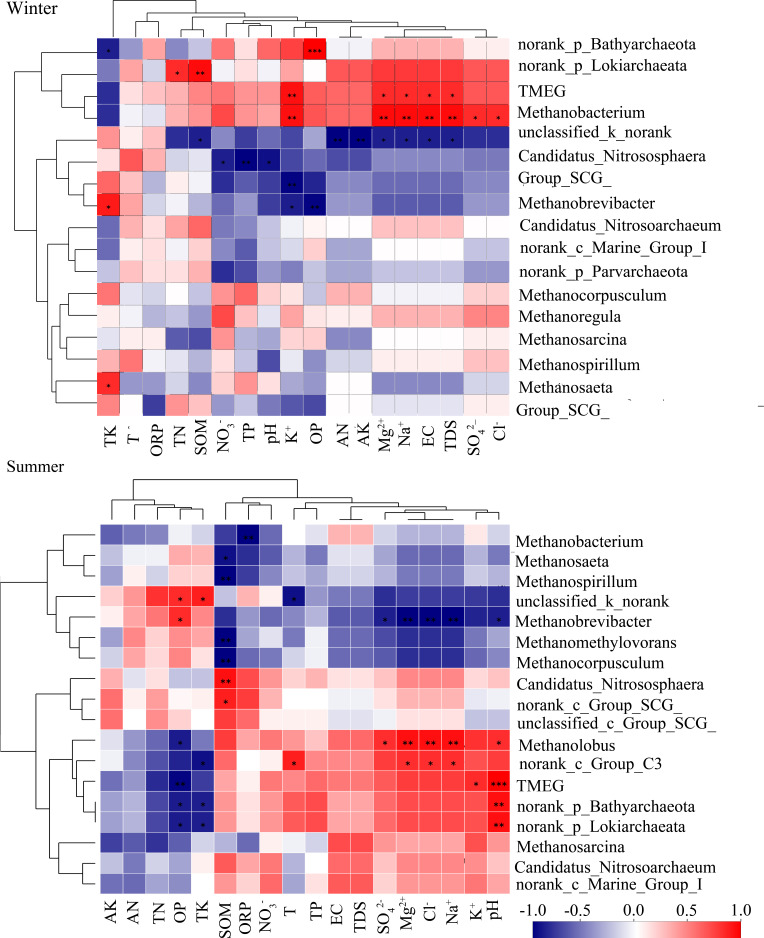
Heat map of environmental factor correlations of archaea. The significant level was symbolled as: *: *p* ≤ 0.05, **: *p* ≤ 0.01, ***: *p* ≤ 0.001. Red represented positive correlation, blue represented negative correlation, the darker the color, the greater the correlation. The range of positive and negative correlation coefficients is −1 to 1. TP: total phosphorus; T: temperature; SOM: soil organic matter; EC: electrical conductivity; TDS: total dissolved solid; AN: alkaline nitrogen; ORP: oxidation-reduction potential; PS: particle size; TN: total nitrogen; OP: Olsen phosphorus; TK: total potassium; AK: alkaline potassium.

Terrestrial_Miscellaneous_Gp_TMEG (TMEG), Lokiarchaeota, *Methanobacterium* were more active in the receiving river reach. TMEG and Lokiarchaeota were newly emerged archaea at FR03. These two archaea were significantly positively correlated with concentrations of SO_4_^2−^, Cl^−^, Na^+^ and K^+^, EC, and TDS in winter (Figure 5), while in summer they were significantly positively correlated with pH and significantly negatively correlated with OP content (Figure 3b). The abundance of TMEG and Lokiarchaeota was more than 2% at FR03, and it was close to 10% at FR04 (Figure 3b). *Methanobacterium* belongs to the hydrogen-nutrient methanogenic archaea and survives in a strict anaerobic environment [71]. The optimum growth temperature of the warm species was 37–45 °C, which were never reached in winter [72]. The concentration of nitrate and sulfate was greatly increased after the injection of the Yellow River water, which could provide abundant nitrogen and sulfur sources for *Methanobacterium*. *Methanobacterium* was most sensitive to the above environmental factors and was highly correlated with them, and it stably survived in the receiving river reach (average abundance of 1.3%). The correlations between the three above-mentioned archaea and various environmental factors were similar. Thus, these three archaea were clustered into one cluster.

For bacteria, Caldilineaceae, Verrucomicrobiaceae, *Novosphingobium,* and OPS_17 were unsuitable for changes caused by water transfer and reduced or even disappeared in the receiving river reach (Figure 3a). Caldilineaceae were significantly positively correlated with the contents of TN, AN, and TK (Figure 4). The contents of TN, AN, and TK decreased in summer by about 44%, 54%, and 16% of that in the water-receiving reach, respectively. Verrucomicrobiaceae was often used as an electron donor for biofuel cells [73]. Perhaps because of this property, Verrucomicrobiaceae was inhibited in water-receiving reach where the ORP decreased due to water transfer. This indicated an increase in e-donors and more competition between these kinds of microorganisms. *Novosphingobium*, as parthenogenic anaerobic bacteria, would be inhibited under a highly dissolved oxygen concentration environment [74]. Water transfer caused the river level to rise and flow faster, creating good water circulation conditions. Thus, the water-substrate interface was more likely to form a high dissolved oxygen environment. OPS_17 was significantly negatively correlated with OP and K^+^, which might be the reason why it was inhibited in the water-receiving reach. After water transfer, both OP content and K^+^ concentration increased obviously. For archaea, *Methanospirillum*’s abundance was relatively low at FR03 (2.05%) and was not detected at other downstream sampling sites. *Methanospirillum*’s dependence on environmental factors was contrary to that of TMEG, Lokiarchaeota, and *Methanobacterium* (Figure 5). The abundance of *Nitrosoarchaeum* at FR03 was reduced by 68.9%, compared with the upstream and downstream (Figure 3b), and the correlation between *Nitrosoarchaeum* and various environmental factors did not reach a significant level. The *Nitrosoarchaeum* participates in the process of nitrification and nitrosation of ammonia in environments [75]. The concentration of NO_3_^−^ at FR03 in winter was 19.8 mg/L, which was 46% and 5% higher than that of the upstream and downstream sampling sites. The obvious change of abundance of *Nitrosoarchaeum* might be related to nitrate at FR03, where nitrate concentration was greatly increased. In conclusion, more bacteria were inhibited after water diversion than archaea. At the same time, many bacteria were active downstream of water transfer inlet, and therefore, the bacterial flora replacement was more obvious than that of the archaea.

Some bacteria and archaea floras appeared only in the water-receiving river. In summer, the new bacteria taxa, including Subgroup_6, *Desulfocapsa*, SC-I-84, Bacteroidetes_VadinHA17, *Sulfuritalea*, KD4-96, and *Thiobacillus*, replaced almost all bacteria taxa with an abundance of more than 1%, except those in the core flora in the FR03. In winter, the new bacteria taxa with an abundance of more than 1%, including Bacteroidetes_VadinHA17, *Fusibacter,* and Dracoribacteriaceae, also appeared in the FR03, obviously less than those in summer. At the same time, TMEG, Lokiarchaeota, and Marine_Group_I in summer, and TMEG, Lokiarchaeota, *Methanobacterium*, and *Methanospirillum* in winter were the new archaea with an abundance of more than 1% in the sediment of FR03, where the Yellow River water was poured into.

In conclusion, bacteria and archaea that successfully and unsuccessfully adapted to the water environment of water receiving reach, and new bacteria and archaea that emerged through water diversion, were sensitive to DO, ORP, salinity, and nutrients. The composition and quantity of microbial functional groups are directly related to water quality characteristics, nutrients levels, and their transformation [76,77]. Water salinity provides a wide range of electron acceptors and electron donors for microbial growth, metabolism, and reproduction [78]. The higher nutrient concentration in the water environment is conducive to the survival of bacteria and archaea that use and break down nutrients, most of which are anaerobic or partly anaerobic. Therefore, after water diversion, although the water flow was accelerated and good water circulation brought a stronger oxidative environment, anaerobic and facultative anaerobic decomposition processes could proceed due to high nutrient concentrations. During this period, the community composition of anaerobic and facultative anaerobic microorganisms resulted in a turnover [79,80]. Under the influence of water transfer, the EC value and main ion content of the river in the study area increased drastically (Table 1), and the contents of TP and OP in the sediment increased significantly (Table 1). Thus, water transfer causes turnover of bacterial and archaeal communities by drastically changing the salinity and nutrient concentration of overlying water.

### 3.4. Different Responses of Bacterial and Archaeal Core Floras to Seasonal Variations

**Figure 6:**
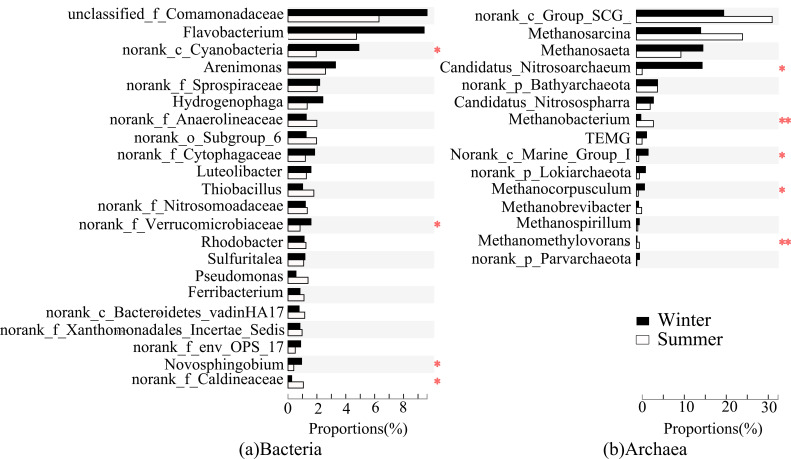
Significance test for bacterial (**a**) and archaeal (**b**) differences due to seasonal variation. The significant level was symbolled as *: *p* ≤ 0.05, **: *p* ≤ 0.01.

There were significant seasonal variations in the abundance of bacteria and archaea. Generally, 1057 bacterial genera and 58 archaea genera were detected in the study area, and 8.6% of the archaea had significant seasonal variation, which was higher than the percentage of 0.4% of the bacteria. Among the bacterial communities, Cyanobacteria, Verrucomicrobiaceae, *Novosphingobium*, and Caldilineaceae were significantly affected by seasonal changes (*p* < 0.05, Figure 6). Except for Caldilineaceae, the other bacterial taxa’s abundances were low in summer and high in winter. Seasonal changes in bacteria were primarily related to the number of nutrients in sediments. *Novosphingobium* had a significant negative correlation with TN and SOM (Figure 4). With SOM decreasing by 36% in winter, the abundance of this strain was significantly higher in winter than in summer. There was a significant positive correlation between Caldilineaceae and TN, AN, TK in summer. With the contents of TN and AN increasing by 28% and 50% in summer, the abundance of this strain was significantly higher in summer than that in winter. Among the archaeal communities, the abundances of *Nitrosoarchaeum* (*p* < 0.05), *Methanocorpusculum* (*p* < 0.05), and *Methanomethylovorans* (*p* < 0.01) were significantly affected by seasonal changes (Figure 6). The abundance of *Nitrosoarchaeum* was much higher in winter than in summer. It had an abundance of more than 10% at FR01–FR04 in January but only appeared at FR02 and FR03 in June with a much smaller abundance. *Methanomethylovorans* were detected only in summer, and their abundance was high at FR05 (5.0%) and FR06 (9.2%).

**Figure 7:**
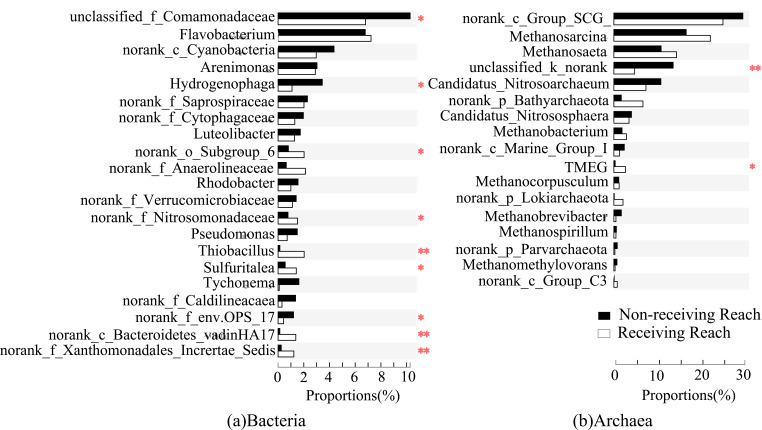
Significance test for bacterial (**a**) and archaeal (**b**) differences due to water diversion. The significant level was symbolled as *: *p* ≤ 0.05, **: *p* ≤ 0.01.

The newly emerged bacteria and archaea in the water-receiving reach also had obvious seasonal changes. For bacteria, the seven new taxa of bacteria (Subgroup_6, *Desulfocapsa*, SC-I-84, Bacteroidetes_VadinHA17, *Sulfuritalea*, KD4-96, and *Thiobacillus*) that were newly emerging in the water-receiving reach were more active in summer than in winter, showed similar correlation with environmental factors, and were classified into one cluster (Figure 4). Among them, Anaerolineaceae, *Thiobacillus*, and Subgroup_6 had more than 4% abundance at FR04 in summer (Figure 3a). In addition to the influence of EC, ion concentration, and nutrient contents in sediments, the newly emerged bacteria in the water-receiving reach were also significantly affected by water temperature (Figure 4), and the abundance increased with increasing temperature. For archaea, the abundance of the newly emerged *Methanobacterium* and Marine_Group_I in the water-receiving reach were significantly different in winter and summer (*p* < 0.05, Figure 7). The adaptability of Marine_Group_I to temperature was different from *Methanobacterium*. Marine_Group_I could not adapt to higher temperatures in summer, which was the same result as the previous studies [81,82]. At the same time, Marine_Group_I have a very close genetic relationship and genetic composition with deep-sea archaea groups, adapting to a high ion concentration environment [83]. The water diversion caused a sudden and substantial increase in the EC value of the river at FR03, which stimulated the abundance of Marine_Group_I to increase to 2.1% even in summer (Figure 3b). In addition, TMEG, Lokiarchaeota, and *Methanospirillum* were significantly reduced in summer.

### 3.5. Differences in Changes of Microbial Diversity and Community Structure

Microbial communities have microbial functional diversity [18]. Changes in dominant flora and their interaction with other flora affected the abundance, diversity, community structure, and stability of microbial ecosystems [84], resulting in corresponding changes in the microbial community’s function. Studies have shown that areas with dramatic changes in the physical and chemical environment could lead to increased abundance and diversity levels of bacteria and archaea, improving the adaptability of the community by producing more microbial populations [75,76,77,78,79,80,81,82,83,84,85,86,87].

In this case, the abundance and diversity of bacterial and archaeal communities in the river sediments were greatly affected by water transfer. However, archaea showed a different changing trend of the abundance and diversity with bacteria. The quantity and diversity of bacteria were higher in water receiving reach, which caused single peaks of the quantity and diversity of bacteria community along the river (Figure S2). However, the quantity and diversity index of archaea were the largest at FR01 and decreased overall along the flow direction. Although there was a drastic environmental change at FR03, the quantity and diversity of archaeal communities decreased in summer. The Sobs and Shannon diversity indexes of archaea were negatively correlated (*p* < 0.05) with the concentrations of Cl^−^, Na^+^, and K^+^ in summer. However, water diversion raised the ion concentration, which decreased archaeal community diversity.

The dominant position of the core bacterial flora decreased in sediments of the water-receiving reach, while the dominant position of the core archaeal flora increased. This was the most obvious difference in the responses of bacterial and archaeal communities to water transfer. The average relative abundance of the core bacterial flora in the sediments of the normal reach in winter and summer was 33.9% and 21.5%, respectively, while that of the water-receiving reach was 29.4% and 20.4%, respectively. The average relative abundance of the core archaeal flora in the sediments of the normal river reach in winter and summer was 79.2% and 75.6%, respectively, while that of water-receiving reach was 89.3% and 83.8%, respectively. Among bacteria, the abundances of Comamonadaceae and *Hydrogenophaga* were significantly lower (*p* < 0.05) in the core bacterial flora in sediments of the water-receiving reach (Figure 6). These two taxa belong to the class of Betaproteobacteria, and hence, they were grouped into one cluster due to their similar correlation with environmental factors (Figure 4). In winter, Comamonadaceae and *Hydrogenophaga* were significantly negatively correlated with TN, SOM, AN, AK of sediments, and EC and Na^+^ of river water. There was a significant positive correlation with TK in summer. Water transfer caused TN, SOM, AN, and AK in sediments to increase by 88%, 58%, 156%, and 125%, respectively, while TK decreased by 16% in summer (Table 2). Therefore, the abundances of Comamonadaceae and *Hydrogenophaga* in sediments were significantly reduced as a result of water transfer. *Hydrogenophaga* disappeared downstream of FR03 during the summer. The water transfer changed the chemical compositions of the river water and the nutrient contents of the sediments, which affected the decrease of the abundance of the core bacterial flora in sediments. Among archaea, the abundance of Bathyarchaeota in the core archaeal flora was significantly increased (*p* < 0.05, Figure 6). Bathyarchaeota ranging from land to ocean has both degradation of refractory organic compounds, autotrophic synthesis of acetic acid using inorganic carbon, and functions involved in methane metabolism [88]. A significant negative correlation between Bathyarchaeota and TK was found in the study area (Figure 5). The content of TK in sediments of the water-receiving reach was decreased by 5% and 16% in winter and summer, respectively. This offered an explanation about the adaption of Bathyarchaeota to the environmental changes brought by water transfer.

**Figure 8:**
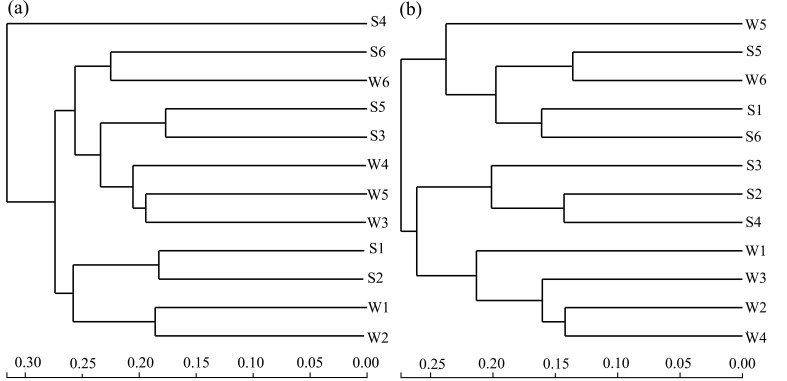
Hierarchical clustering of bacteria (**a**) and archaea (**b**). The length of the scale represents the differences between the community composition structures of the samples. The longer the distance is, the greater the difference becomes. The sequencing results of the sampling points FR01–FR06 in winter and summer were labeled as W1–W6 and S1–S6, respectively.

The water transfer is the main factor for the changes of bacterial community structure in the river sediments, while seasonal changes are the main factors for the changes of archaeal community structure. Bacterial communities in all sediment samples could be clustered into three clusters (Figure 8a). Except for S4, the remaining samples are divided into two clusters by the normal river reach and the receiving river reach. Within the two clusters, samples are further divided according to the two seasons. The clustering results suggested that water transfer imposed more influences on the changes of bacterial community structure in the river sediments than the changing seasons. The archaeal community samples can be clustered into two clusters (Figure 8b). The community structure of FR01 was similar to that of FR05 and FR06, and S1, S5, W5, S6, and W6 are divided into the first cluster. The dominant archaea of FR02–FR04 had the same characteristics to form the second cluster. In W1, due to the higher abundance of *Nitrosoarchaeum* (Figure 3b), it was divided into the second cluster. The results of this classification did not separate the winter and summer samples, nor did they separate the normal river reach and water-receiving reach. In the second cluster, samples from FR02, FR03, and FR04 impacted most by water transfer were further divided into two subclusters according to the two seasons, indicating that the changing seasons played a greater role in changes of the archaeal community structure in sediment than water transfer.

Both bacterial and archaeal communities showed resistance and stability to a certain degree in the studied river sediments. Additionally, the differences in the stability of the resistance between the bacterial and archaeal communities exist. The abundance and diversity of bacterial OTUs in the river sediments were much higher than those of archaea (Figure S2). Although bacteria are sensitive to environmental changes, the abundant species and complex community structure of the bacterial community made it resistant to external disturbances. The species composition and structure of the archaeal community were relatively simple compared with bacteria. The proportion of core archaeal flora abundance was 67.9% higher than that of bacteria. In front of external environmental changes and stress, archaeal communities are less but more stable in performance. However, under the pressure of drastic environmental changes caused by water transfer in the short term, the abundance and diversity of archaea still showed a decreasing trend, although more archaeal species were produced to adapt to the environmental changes.

This pattern exists in numerous related studies. The temperature was the main factor driving the seasonal changes of the bacterial community [29,30]. Archaeal communities in the Bay of Banyuls area were strongly influenced by terrestrial sources, but changes in marine conditions played a more important role in the construction of bacterial communities [28]. In arid saline land, the abundance of archaea was abnormally much higher than that of bacteria [31]. These seem to be explained by the fact that bacteria are more adapted to slowly changing environments, such as changes in seawater conditions and changes in temperature due to seasonal variations. Archaea, on the other hand, show advantages in drastically changing environmental conditions, such as the turnover of terrestrial sources along the coast that often changes abruptly with the direction of ocean currents, and the salinity of arid saline surface soils that changes rapidly with the alternation of rainy and dry seasons [28,31].

Bacteria have significant advantages in terms of diversity over archaea [89]. From a phylogenetic point of view, the bacterial domain contains more biological lineages than other domains. In the same sample size, the archaea contain species that are smaller than the bacterial range. Consistent with this view, archaea found in many ecosystems, such as seawater [90], hydrothermal vents [91], and subsurface surfaces [92], whereas microbial communities associated with humans [93] are less diverse, which is due to archaea evolving more slowly than bacteria. This might be the result of maintaining a stable expression of a particular phenotypic gene that adapts to extreme environments. Archaea are therefore thought to be the earliest form of life on earth [94]. Although bacteria and archaea are both important forces involved in the elemental biogeochemical cycle, results in this paper found significant differences in their abundance, diversity, community structure, and stability in response to anthropogenic or natural environmental impacts. The mechanism responsible for the difference might be related to differences in their cellular structures, enzyme systems, and branches of evolutionary kinship, which is for further study.

### 3.6. Microbial Interaction

According to the different responses of bacteria and archaea in river sediments to human water transfer activities and natural seasonal changes, bacteria can be divided into two categories, i.e., those mainly affected by water transfer, such as Comamonadaceae, *Hydrogenophaga*, Subgroup_6, Anaerolineaceae, Nitrosomonadaceae, *Sulfuritalea*, *Thiobacillus*, OPS_17, Bacteroidetes_vadinHA17, and Xanthomonadales (Figure 6), and those mainly affected by changing seasons, such as Verrucomicrobiaceae, Cyanobacteria, *Novosphingobium*, and Caldilineaceae (Figure 7). Archaea can be divided into three categories, i.e., those that are mainly sensitive to human activities, such as Bathyarchaeota, TMEG, and Lokiarchaeota (Figure 6), archaea mainly affected by seasonal changes, such as *Nitrosoarchaeum*, *Methanocorpusculum*, and *Methanomethylovorans* (Figure 7), and lastly, those that produce significant responses to both water-transfer activities and seasonal changes, such as the *Methanobacterium* and Marine_Group_I (Figure 3b and Figure 7). Archaea in the river sediments can produce significant responses to water transfer and seasonal changes simultaneously, which is different from bacteria.

**Figure 9:**
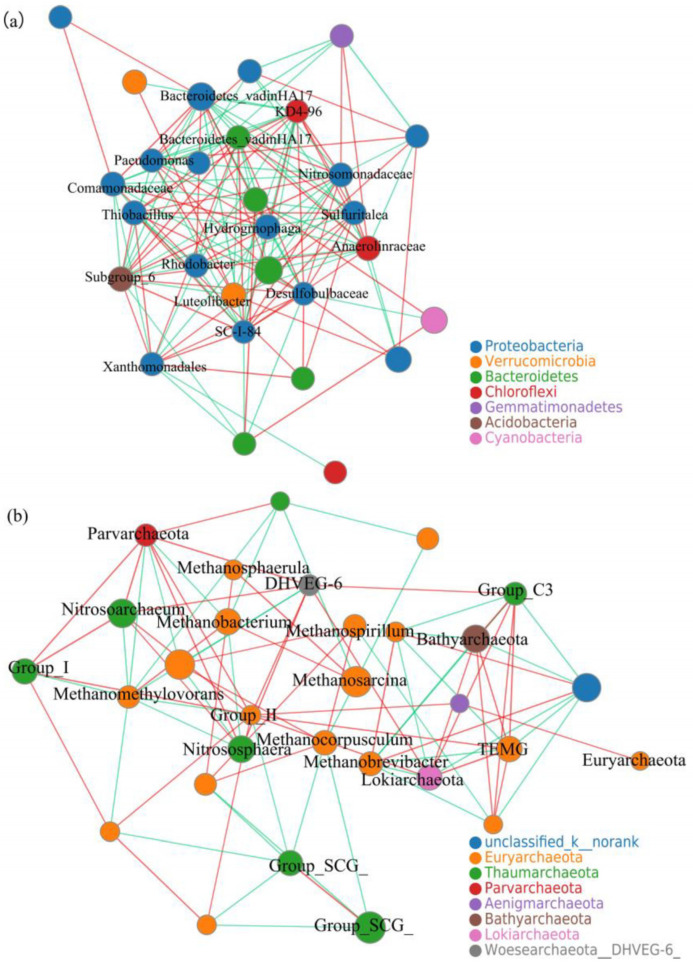
Network analysis of bacteria (**a**) and archaea (**b**). Nodes represent taxa, and the size of the node indicates the relative abundance of the taxon. Different colors of nodes represent different phylum. The red line represents a positive correlation between the two bacteria and the green line represents a negative correlation.

There were fewer interactions between the two classes of bacteria (Figure 9a). In general, the bacteria mainly affected by water transfer are significantly positively correlated with most environmental factors, especially nitrogen, phosphorus, and organic matters in sediments, and EC, TDS, and ion concentrations in river water. However, the bacteria mainly affected by seasonal changes are negatively or weakly correlated with most of the environmental factors (Figure 4). The different correlations with environmental factors provide an explanation for the weak interaction between the two classes of bacteria. In fact, there were only one or two species that had significant interactions with bacteria that are mainly affected by seasonal changes. Therefore, the bacteria mainly affected by the seasonal changes were relatively independent in the community.

The three types of archaea interacted significantly. There were 10 taxa that had significant interaction with *Nitrosoarchaeum* (synthesis with seven taxa and the other three were antagonistic). Similarly, both TMEG and Lokiarchaeota interacted with eight taxa, Marine_Group_I interacted with seven genera, and *Methanobacterium* interacted with four taxa (Figure 9b). In addition, although the abundances of *Methanocorpusculum*, *Methanomethylovorans,* and Woesearchaeota were low, their interactions with other species were strong. They are mostly related to the carbon and nitrogen cycle in environments, and their metabolites and metabolic processes have great influences on other species. Therefore, the archaea with low abundance also played an important role in the archaeal community.

Complex interspecific regulatory mechanisms exist among bacteria. Studies have shown that inter- and intraspecific quorum-sensing (QS), in which microorganisms secrete and sense chemical signaling molecules, is the main pathway regulating microbial interactions and can regulate gene expression based on population density [95]. This mechanism was able to avoid the overlap of bacterial ecological niches [96]. Thus, in this study, the response patterns between bacteria that responded significantly to the season and bacteria that responded significantly to water regulation differed between environmental factors, and the two groups of bacteria were independent of each other. In contrast, there was no obvious ecological niche separation in archaea. At present, the mechanism of interspecific interactions of archaea is not clear [97,98]. When scientists study interspecific interactions between bacteria and archaea, they often assume that the two have similar regulatory mechanisms [99]. However, these results suggested that the interspecies regulation mechanisms of archaea and bacteria may not be the same.

## 4. Conclusions

The water diversion project, as an anthropogenic short-term drastic environmental disturbance, significantly altered EC, ORP, and salinity of water, as well as nutrient content in the sediment through allochthonous inputs and hydrodynamic disturbances. Seasonal changes, as a long-term slow natural variation, had a significant effect on water temperature (*p* < 0.05).

Bacteria and archaea responded differently to the water transfer project and seasonal changes. The dominance of core bacterial flora decreased, and that of core archaeal flora increased in sediments of water receiving reaches. This study revealed that the reason is that bacteria and archaea had different stability and reversal mechanisms in response to natural and anthropogenic disturbances. Bacterial communities in sediment were more sensitive to anthropogenic and naturally induced environmental changes than archaeal communities. However, the rich species composition and complex community structure of bacterial communities made them showed stronger resistance than archaeal communities to long-term external disturbances such as seasonal changes. Archaea had a simple community composition, but their insensitivity to environmental changes made them more stable than bacteria, especially in the face of short-term drastic environmental disturbances, such as water diversion projects.

In addition, the results of this study indicated that bacteria and archaea had different interspecific regulatory mechanisms. This phenomenon has guiding implications for related studies.

## Figures and Tables

**Table 1:** Physicochemical features of the river water and sediment.

		January		June	
		Normal River Reach	Water-Receiving Reach	Normal River Reach	Water-Receiving Reach
Water	T (°C)	1.4 ± 1.0	1.2 ± 0.5	19.6 ± 0.1 *	21.9 ± 0.6 *
	EC (μs/cm)	535.0 ± 28.0 **	1257.0 ± 27.0 **	436.0 ± 34.0 **	1205.0 ± 32.0 **
	ORP (mv)	410.0 ± 55.2	366.0 ± 15.0	209.4 ± 26.3	199.2 ± 22.5
	TDS (g/L)	0.3 ± 0.0 **	0.8 ± 0.0 **	0.3 ± 0.2 **	0.8 ± 0.0 **
	NO_3_^−^ (mg/L)	12.0 ± 1.7 *	19.7 ± 0.5 *	12.4 ± 2.6	11.4 ± 1.7
	SO_4_^2−^ (mg/L)	70.1 ± 6.7**	205.5 ± 3.7 **	121.6 ± 0.9 *	400.9 ± 116.3 *
	Cl^−^ (mg/L)	6.6 ± 0.8 **	132.3 ± 3.8 **	7.0 ± 0.4 **	150.9 ± 67.6 **
	Na^+^ (mg/L)	6.7 ± 0.6 **	121.1 ± 3.8 **	8.4 ± 1.2 **	152.2 ± 3.9 **
	K^+^ (mg/L)	1.8 ± 0.0	3.9 ± 0.0	2.4 ± 0.3	4.9 ± 0.6
	Mg^2+^ (mg/L)	15.5 ± 0.7 **	37.0 ± 0.6 **	16.8 ± 1.5 *	41.0 ± 0.6 *
	Ca^2+^ (mg/L)	63.7 ± 0.2	67.6 ± 1.6	76.3 ± 7.0	65.2 ± 3.7
Sediment	PS (μm)	105.0 ± 33.0	53.0 ± 18.0	247.0 ± 147.0	116.0 ± 61.0
	pH	7.8 ± 0.0	8.1 ± 0.2	8.4 ± 0.4	8.9 ± 0.1
	SOM (g/kg)	14.7 ± 1.9	23.2 ± 8.3	11.9 ± 3.7	16.0 ± 4.2
	TN (g/kg)	4.4 ± 0.0	8.3 ± 0.1	11.3 ± 0.0 *	6.4 ± 0.0 *
	AN (mg/kg)	33.0 ± 2.0 *	84.3 ± 23.1 *	160.0 ± 29.0 *	73.0 ± 8.5 *
	TP (mg/kg)	464.5 ± 36.5	578.5 ± 45.3	530.0 ± 50.0	589.3 ± 44.4
	OP (mg/kg)	5.5 ± 0.5	10. 3 ± 3.9	17.0 ± 4.0	7.8 ± 1.8
	TK (g/kg)	28.3 ± 0.0	27.0 ± 0.2	28.2 ± 0.2	23.5 ± 0.1
	AK (mg/kg)	48.5 ± 6.5 *	109.8 ± 24.3 *	69.0 ± 1.0	78.0 ± 15.5

The normal river reach includes sites of FR01 and FR02. The water-receiving reach includes sites of FR03–FR06. * indicates the difference between values from different river reaches is significant at the *p* < 0.05; ** indicates difference is significant at the *p* < 0.01. PS: particle size; SOM: soil organic matter; TN: total nitrogen; AN: alkaline nitrogen; TP: total phosphorus; OP: Olsen phosphorus; TK: total potassium; AK: alkaline potassium.

**Table 2:** Operational taxonomic units (OTUs) number and α-diversity index of bacteria and archaea.

Sample	Season	OTUs		Shannon Index		Chao Index		Coverage Index	
		Bacteria	Archaea	Bacteria	Archaea	Bacteria	Archaea	Bacteria	Archaea
FR01	Winter	2822	590	6.06	3.56	3768.44	733.13	0.98	0.99
	Summer	2149	504	5.94	3.32	2859.82	602.89	0.99	0.99
FR02	Winter	3146	512	6.51	3.10	4103.01	610.25	0.98	0.99
	Summer	2884	456	6.42	3.17	3857.02	619.43	0.98	0.99
FR03	Winter	3631	582	6.76	3.26	4722.66	789.59	0.97	0.99
	Summer	3549	392	6.86	2.95	4470.78	553.25	0.98	0.99
FR04	Winter	3659	363	6.77	3.08	4717.44	462.13	0.98	0.99
	Summer	3602	237	6.92	3.03	4479.15	326.44	0.98	0.99
FR05	Winter	3441	288	6.51	2.42	4513.04	380.57	0.98	0.99
	Summer	3737	298	7.01	2.94	4746.85	385.15	0.98	0.99
FR06	Winter	2708	335	5.79	2.89	4044.36	450.93	0.98	0.99
	Summer	2303	348	5.76	3.06	3261.68	461.56	0.98	0.99
Average	Winter	3234.5	445	6.40	3.05	4311.49	580.51	0.98	0.99
	Summer	3037.3	372.5	6.48	3.08	3945.88	491.45	0.98	0.99

## Data Availability

Not applicable.

## Supplementary Materials

The following are available online at https://www.mdpi.com/article/10.3390/microorganisms9040782/s1, Figure S1: Sobs, Shannon and Coverage curves of bacteria (A) and archaea (B), Figure S2: Shannon (a) and Sobs (b) index line graphs for bacteria and archaea, Figure S3: Evolutionary relationship of bacteria (a) and archaea (b).
