# Supplementary material for: Different Responses of Bacterial and Archaeal Communities in River Sediments to Water Diversion and Seasonal Changes
Source: Microorganisms. 2021 Apr 8;9(4):782. doi: 10.3390/microorganisms9040782 (PMC8068392; doi:10.3390/microorganisms9040782)
Supplement: Supplementary file 1 [file microorganisms-09-00782-s001.pdf]

microorganisms

Different Responses of Bacterial and Archaeal Communities in River Sediments to Water Diversion and Seasonal Changes

Jiali Lv [1,2,3], Yangdan Niu [1], Ruiqiang Yuan [1,*] and Shiqin Wang [2]

[1] School of Environment and Natural Resources, Shanxi University, Taiyuan 030006, China; lvjiali19@mails.ucas.ac.cn (J.L.); niuyangdan@tongji.edu.cn (Y.N.); rqyuan@sxu.edu.cn (R.Y.)
[2] Key Laboratory of Agricultural Water Resources Research, Innovation Academy for Seed Design, Center for Agricultural Resources Research, Institute of Genetics and Developmental Biology, Chinese Academy of Sciences, Shijiazhuang 050021, China; sqwang@sjziam.ac.cn (S.W.).
[3] Sino-Danish College, University of Chinese Academy of Sciences, Beijing 101408, China
* Correspondence: rqyuan@sxu.edu.cn

Citation: Lv, J.; Niu, Y.; Yuan, R.; Wang, S. Different Responses of Bacterial and Archaeal Communities in River Sediments to Water Diversion and Seasonal Changes. *Microorganisms* **2021**, *9*, 782. https://doi.org/10.3390/microorganisms9040782

Academic Editor: Siqing Liu

Received: 23 March 2021
Accepted: 7 April 2021
Published: 8 April 2021

Publisher's Note: MDPI stays neutral with regard to jurisdictional claims in published maps and institutional affiliations.

Copyright: © 2021 by the authors. Licensee MDPI, Basel, Switzerland. This article is an open access article distributed under the terms and conditions of the Creative Commons Attribution (CC BY) license (http://creativecommons.org/licenses/by/4.0/).

1.1.α-. Diversity of Bacteria and Archaea

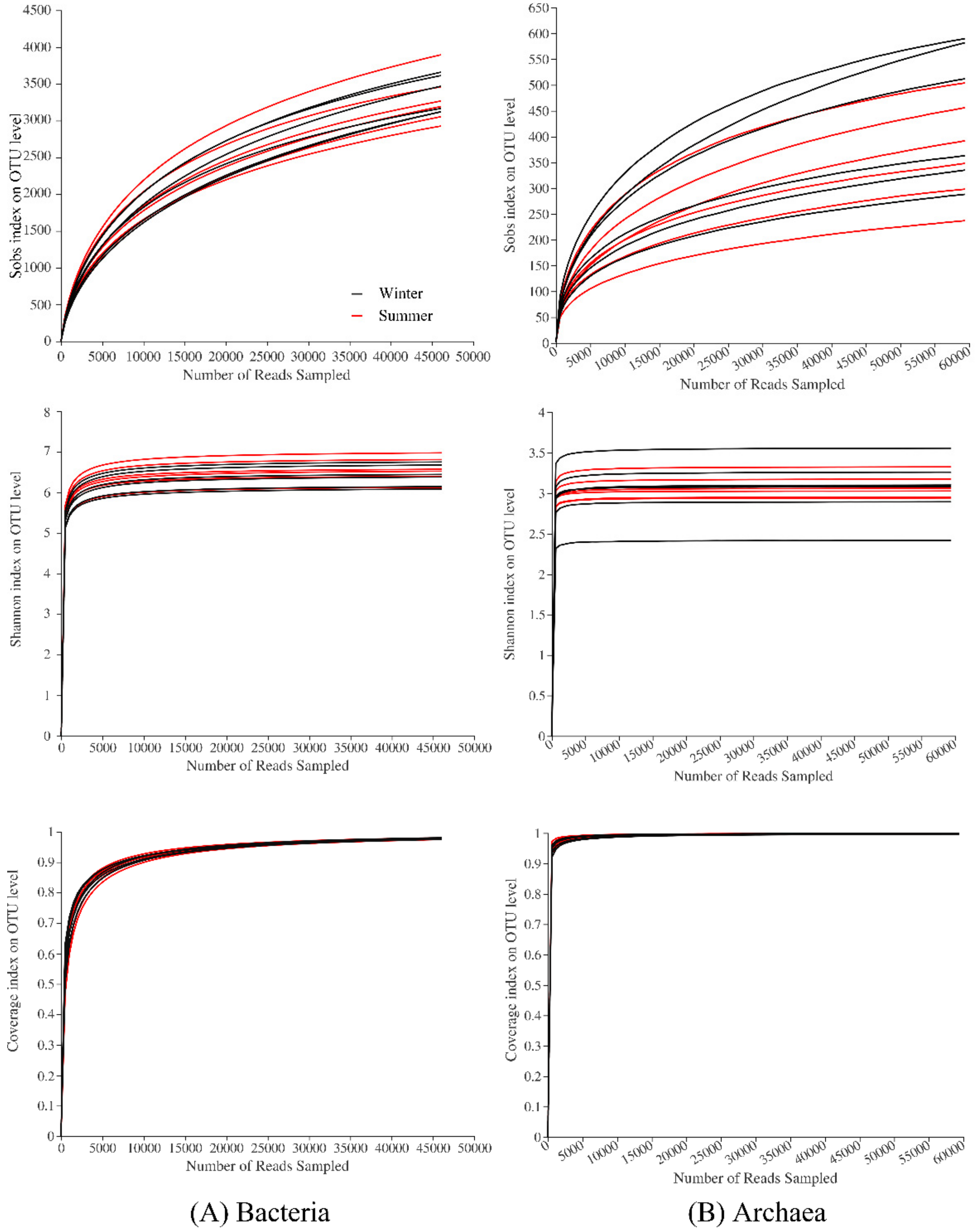

Figure 1. Sobs, Shannon and Coverage curves of bacteria (A) and archaea (B).

The rarefaction curve of Sobs, Shannon and Coverage diversity indexes gradually became smooth with the increase of sequencing depth (Figure S1). The sequencing depth is 30,000. At such depth, the Sobs, Shannon and Coverage diversity indexes for the observed OTUs was stable, indicating that the sequencing data volume was large enough to reflect microbial diversity information in the samples.

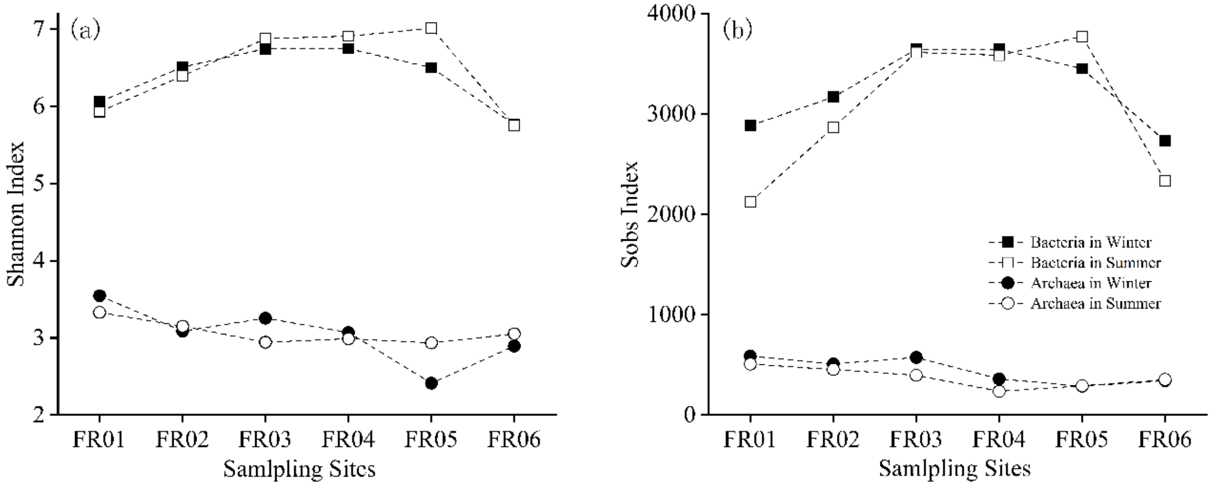

Figure 2. Shannon (a) and Sobs (b) index line graphs for bacteria and archaea.

The number of bacteria showed a pattern of rising and then falling by water transfer, while the number of archaea has been slowly declining. The number of bacterial OTUs fluctuate more in the summer, while that of archaea fluctuate more in the winter.

1.2. Evolution of Bacteria and Archaea

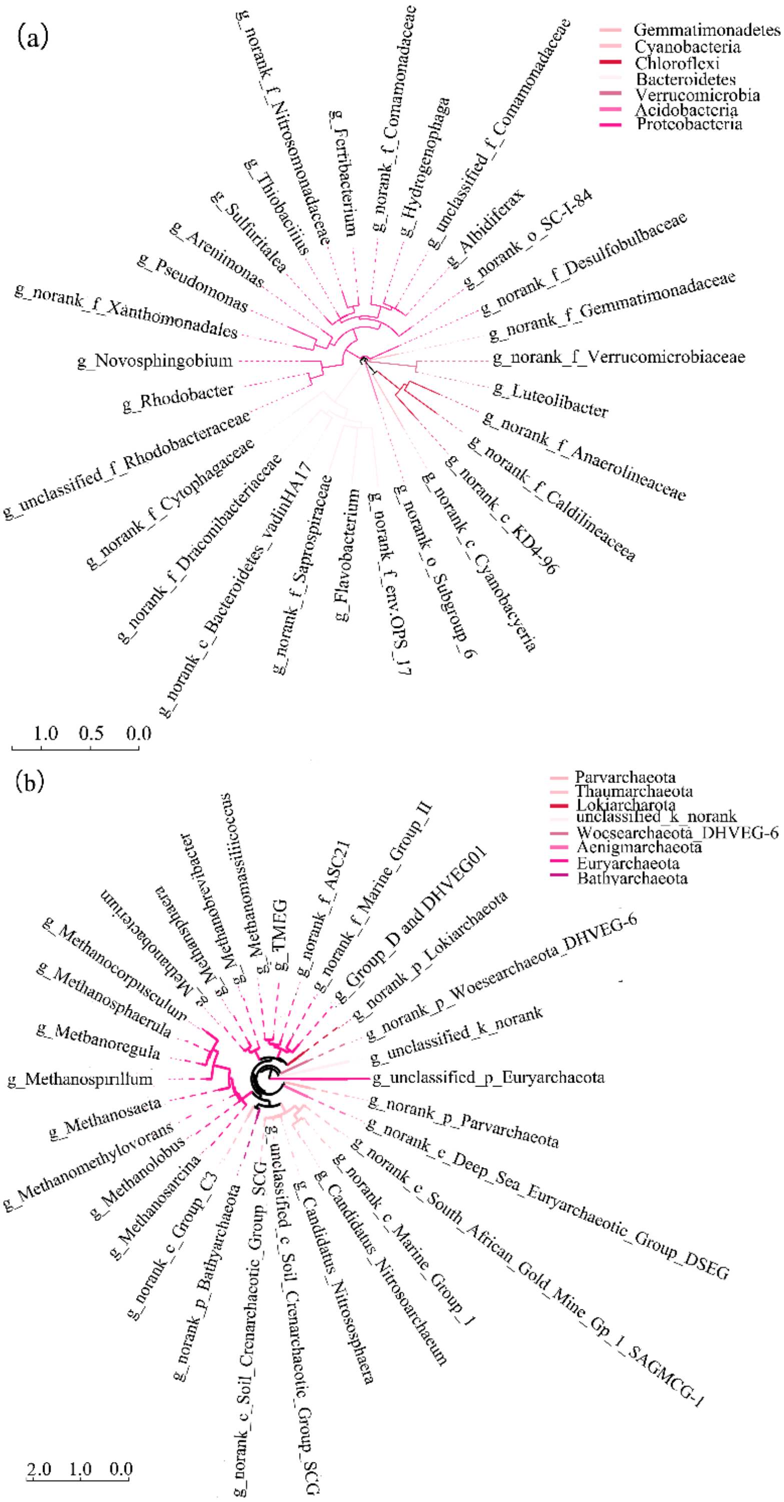

Figure 3. Evolutionary relationship of bacteria (a) and archaea (b). The color of the tree branches represents the phylum to which the bacteria or archaea belong.

Similarities and differences between species are quantified and visualized by distance. The shorter the distance the closer the affinity between species.
